# The Netherlands study of depression in older persons (NESDO); a prospective cohort study

**DOI:** 10.1186/1756-0500-4-524

**Published:** 2011-12-05

**Authors:** Hannie C Comijs, Harm W van Marwijk, Roos C van der Mast, Paul Naarding, Richard C Oude Voshaar, Aartjan TF Beekman, Marjolein Boshuisen, Janny Dekker, Rob Kok, Margot WM de Waal, Brenda WJH Penninx, Max L Stek, Johannes H Smit

**Affiliations:** 1 Department Psychiatry/EMGO Institute for Health and Care Research, VU University Medical Center/GGZinGeest, Amsterdam, The Netherlands; 2 Department of General Practice and EMGO Institute for Health and Care Research, VU University Medical Center, Amsterdam, The Netherlands; 3 Department of Psychiatry, Leiden University Medical Center, Leiden, The Netherlands; 4 Department of Old-age Psychiatry, GGNet, Apeldoorn/Zutphen, The Netherlands; 5 University Center of Psychiatry & Interdisciplinary Center of Psychiatric Epidemiology, University Medical Center Groningen, Groningen, The Netherlands; 6 Department of Psychiatry, Radboud University Nijmegen Medical Center, Nijmegen, The Netherlands; 7 Department Psychiatry/EMGO Institute for Health and Care Research, VU University Medical Center, Amsterdam, The Netherlands; 8 Lentis, Dignis, Groningen, The Netherlands; 9 Department of General Practice, University Medical Center Groningen, Groningen, The Netherlands; 10 Department of Old-age Psychiatry, Parnassia/BAVO groep, The Hague, The Netherlands; 11 Department of Public Health and Primary Care, Leiden University Medical Center, Leiden, The Netherlands; 12 Department Psychiatry, EMGO Institute for Health and Care Research and Institute for Neurosciences, VU University Medical Center, Amsterdam, The Netherlands; 13 Department of Old-age Psychiatry, GGZinGeest, Amsterdam, The Netherlands; 14 Department Psychiatry/EMGO Institute for Health and Care Research/Institute for Neurosciences, VU University Medical Center and GGZ inGeest, Amsterdam, The Netherlands; 15 Department Psychiatry, VUMC, A.J. Ernststraat 1187, 1081 HL Amsterdam, The Netherlands

## Abstract

**Background:**

To study late-life depression and its unfavourable course and co morbidities in The Netherlands.

**Methods:**

We designed the Netherlands Study of Depression in Older Persons (NESDO), a multi-site naturalistic prospective cohort study which makes it possible to examine the determinants, the course and the consequences of depressive disorders in older persons over a period of six years, and to compare these with those of depression earlier in adulthood.

**Results:**

From 2007 until 2010, the NESDO consortium has recruited 510 depressed and non depressed older persons (≥ 60 years) at 5 locations throughout the Netherlands. Depressed persons were recruited from both mental health care institutes and general practices in order to include persons with late-life depression in various developmental and severity stages. Non-depressed persons were recruited from general practices. The baseline assessment included written questionnaires, interviews, a medical examination, cognitive tests and collection of blood and saliva samples. Information was gathered about mental health outcomes and demographic, psychosocial, biological, cognitive and genetic determinants. The baseline NESDO sample consists of 378 depressed (according to DSM-IV criteria) and 132 non-depressed persons aged 60 through 93 years. 95% had a major depression and 26.5% had dysthymia. Mean age of onset of the depressive disorder was around 49 year. For 33.1% of the depressed persons it was their first episode. 41.0% of the depressed persons had a co morbid anxiety disorder. Follow up assessments are currently going on with 6 monthly written questionnaires and face-to-face interviews after 2 and 6 years.

**Conclusions:**

The NESDO sample offers the opportunity to study the neurobiological, psychosocial and physical determinants of depression and its long-term course in older persons. Since largely similar measures were used as in the Netherlands Study of Depression and Anxiety (NESDA; age range 18-65 years), data can be pooled thus creating a large longitudinal database of clinically depressed persons with adequate power and a large set of neurobiological, psychosocial and physical variables from both younger and older depressed persons.

## Background

The prevalence of major depression in older persons living in the community ranges from 1-5%. [1] Rates of depressive disorders are substantially higher among specific populations of older persons, ranging from 5-10% in medical outpatients to 14-42% in residents of long-term care facilities. [1] The prognosis of late-life depression is often poor. [2] It appears to have a chronic course and higher relapse rates compared to early-life depression [3-6] and co morbidity with cognitive decline and somatic diseases is higher than in depression in younger adults. [7-9] In addition, in late-life depression co morbidity with other psychiatric disorders, especially anxiety disorders is high, [10-13] and leads to longer time to remission as well as higher recurrence rates [14].

These findings suggest that the risk factors for late-life depression and its course may change during lifetime. According to the Stress-Vulnerability Model [15], the interaction of three factors is responsible for the development of depression and its course over time: vulnerability, stress and protective factors. However, vulnerability factors, stressors and protective factors may change over time, and the extent to which these factors interact and influence mental health may change as well. Former studies suggest that personality, genetic factors as well as problems at work and in family relationships may play a larger role in the onset of depression at early age, whereas late-life depression has been hypothesized to be more strongly associated with frailty-associated processes and neurodegenerative biological abnormalities. [16,17] It is shown that psychosocial as well as biological factors play a role in (the course of) late-life depression and its co morbidities. [4,18-23] Several of these determinants are supposed to be common in older adults, such as loneliness and losses in social environment, [24] systemic low-grade inflammation [25] and functional and cognitive limitations. [26] In addition, some of these determinants seem to function differently in older compared to younger adults, such as the hypo activation instead of hyper activation of biological stress-regulation systems; the hypothalamic-pituitary-adrenal (HPA)-axis and the autonomic nervous system (ANS) [27-32].

Many of these determinants for late-life depression are also interrelated, for instance, dysregulation of stress regulating systems in turn give rise to dysregulation of the immune system and to altered vascular and metabolic processes, which may ultimately have deleterious effects on the cardiovascular system, cognition and mood regulation, [33,34] although studies in this field show conflicting results. [35] More importantly, not only can somatic diseases be a contributing or etiological factor to late-life depression, late-life depression itself also increases the risk of the development of new somatic disease, such as cardiovascular disease [23,36-40] and diabetes mellitus [41] and of physical impairment [42].

A pronounced role for biological and neurodegenerative underlying mechanisms in (the course of) late-life depression may also explain the differences that have been found between the symptom profiles of early- and late-life depression. Compared to younger depressed adults, older depressed persons were found to show more psychomotor dysregulation and somatic disturbances such as fatigue and sleep disturbance [43,44] and to have more apathy without the more traditional symptoms of depression [45,46].

To better understand late-life depression, and its unfavourable course and co morbidities, we need longitudinal studies that integrate underlying psychosocial and biological vulnerability and protective mechanisms. Furthermore, it is still questioned in what way late-life depression differs from depression earlier in life. Leaving us with the question whether the concept of late-life depression is similar to that of early-life depression and, thus, to what extent we need differential diagnostic criteria and treatment strategies. To be able to make a direct comparison between early- and late-life depression we need studies that have sufficient numbers of depressed persons in all age ranges. Therefore, we designed the Netherlands Study of Depression in Older Persons (NESDO; http://nesdo.amstad.nl) in which the course of late-life depression and its co morbidities will be followed up in detail in a prospective design. To be able to make a direct comparison between early- and late-life depression, NESDO uses the same design and overlapping instruments as the Netherlands Study of Depression and Anxiety (NESDA). [47] NESDA has collected data from a large cohort of persons with depressive and anxiety disorders aged 18 through 65 years (*N *= 2,981), which makes it possible to directly compare data from the older cohort with the existing data from depressed younger persons from NESDA (*n *= 1,149), thus creating a large longitudinal database of clinically depressed persons with adequate power and a large set of neurobiological, psychosocial and physical variables from both younger and older depressed persons.

### Aims of the study

The first aim for NESDO is to examine the determinants of late-life depression. The second aim is to examine the course, the determinants of the course and the consequences of depressive disorders in older persons. The third aim is to compare the determinants of late-life depression, and the course and its determinants with that of early-life depression.

## Methods

### Sample

Recruitment of depressed older persons took place in five regions in the Netherlands from both mental health care institutes and general practitioners in order to include persons with late-life depression in various developmental and severity stages. Persons with a primary diagnosis of dementia, were suspected for dementia according to the clinician or had a Mini Mental State Examination-score (MMSE) under 18 (out of 30 points) were excluded. Also persons with insufficient command of the Dutch language were excluded. Non-depressed controls were included in order to answer research questions on the aetiology of depression, and were recruited from general practitioners. Inclusion criteria for non-depressed controls were: no lifetime diagnosis of depression or dementia, and good command of the Dutch language. Data collection of the first measurement started in 2007 and was finished in September 2010.

Three hundred and twenty-six persons of the depressed older persons (86.2% of the depressed sample) were recruited from the out- and inpatient clinics of the regional facilities for mental health care: Amsterdam (*n = *113), Leiden (*n = *70), Apeldoorn/Zutphen (*n = *50), Nijmegen (*n = *47) and Groningen (*n = *46). Patients with a primary diagnosis of major depression, dysthymia or minor depression according to DSM-IV criteria [48] and who were mentally able to give written informed consent received verbal and written information about NESDO and were asked to participate. Response rates are only available from Amsterdam, but these may be considered representative for the other mental health institutions. In the region of Amsterdam, 294 persons with a depressive disorder were enrolled to the study. Of these, 291 (99%) were contacted by telephone and 3 (1.0%) could not be reached despite multiple efforts. Of the persons who were approached by telephone, 57 persons (19%) were excluded because of meeting the exclusion criteria: 23 (7.8%) had had no depressive symptoms in the past 3 months, 14 (4.7%) were not able to undergo the interview because of severe mental illness or severe physical or cognitive frailty, and 6 (2.0%) were excluded because of language problems during the phone contact. Of the remaining 234 persons who fulfilled the inclusion criteria, 121 persons (51.3%) refused and 113 (48.7%) were willing to participate in NESDO. Compared with those who refused to participate, persons who participated in the baseline interview were comparable in gender, but were younger (72.0 years versus 74.2 years, p < 0.03).

The primary care patients (*n = *52) and the non-depressed control group (*n = *132) were recruited from 14 general practices (GPs) in the vicinity of Amsterdam, Groningen and Leiden. Depressed persons were referred by the GPs because of depressive complaints (*n = *17), or after screening with the fifteen item version of the Geriatric Depression Scale (GDS-15; *n = *35). [49] A screen positive score on the GDS-15 was defined as a score of 4 or higher. In total, 242 screen positive persons were contacted for a short phone-screen interview of whom 58 (24.0%) fulfilled the criteria for a current depression and the exclusion criteria. Of these, 23 (39.7%) refused to participate in the NESDO study, and 35 persons (60.3%) with a current depressive disorder underwent the baseline assessment. In addition, a random selection of screen-negative persons (*n = *198) were approached for a short phone-screen and those who fulfilled the criteria for a non-depressed control and had sufficient command of the Dutch language were invited to participate in NESDO. Of these, 132 (66.7%) were willing to participate in the study.

### Sample size

Our sample size calculations indicated that for cross-sectional analyses with the baseline NESDO sample and a power of 80% and an alpha of 0.05 we will be able to detect small differences with respect to clinical variables and questionnaires. For the biological determinants we need to extend the sample with persons of 60-65 years from NESDA (depressed persons: *N = *77; normal controls *N = *102), which results in a sample of 455 depressed and 234 non-depressed elderly, to detect moderate effects in the biological measures. When we extend the NESDO sample with younger age groups, the sample size will be suitable to detect even small effects in these biological measures.

For the analysis on the course of depression, we need a minimum of 255 persons to detect an odds ratio of 1.4 in a multiple logistic regression models with a categorical outcome variable (for instance, presence of depression diagnosis yes/no) with a power of 80% and a level of significance of 0.05. In a multiple regression models with a continuous change variable as outcome (for example change in severity of depressive symptoms), with a sample size of 255 and a power of 80% and an alpha of 0.05, we are able to detect a 5% change in the variance. So, the sample size is adequate to demonstrate statistical significances for even small differences. If we would examine change in the outcomes combining the 3 follow-up assessments using a multi-level analyses, our power would even be substantially higher than in the above described examples.

### Assessment

The Stress-Vulnerability Model [15] that suggests that the interaction of vulnerability, stress and protective factors are responsible for the development of depression and its course over time has guided the selection of the instruments. The baseline assessment included internationally accepted, commonly used measures for demographic variables, depression, psychosocial variables, (chronic) stressors, activity of the HPA-axis, (re)activity of the ANS, parameters for inflammation, somatic co morbidity, cognitive functioning and the use medication and health care services (Table 1). The baseline assessment took place in the morning and lasted 3 to 4 hours. It included written questionnaires, an interview, a medical examination, assessment of the ANS (re)activity, collection of blood and instructions for sampling of the saliva. The baseline assessment was mainly performed at the different participating sites. When participants were not able to come to the site, they were interviewed at their homes. Furthermore, when necessary, the assessment was spread over two appointments. After the assessment, the participants were compensated with a small incentive (gift certificate of 15 euro and payment of travel costs) for their time and cooperation. Participants are given feedback about the research program by means of yearly newsletters. Furthermore, there was prompt response to any question or letter from the participants. Cooperation was facilitated by offering the participants a mix of self-report questionnaires, interviews, biological measures and cognitive tests.

**Table 1:** Baseline assessment of NESDO

Topic	Measurement instrument
Demographics: Age, gender, education, SES^a^, religion, partner status, family	Standard questions

Diagnostics	Composite Interview Diagnostic Instrument (CIDI) -version 2.1 World Health Organization (WHO), 1997: depressive and anxiety disorders

Psychopathology	

- Depression, severity	Inventory of Depressive Symptoms (IDS; Rush et al., 1996,

- Apathy, severity	Apathy Scale (Starkstein et al., 1992)

- Anxiety symptoms	Beck Anxiety Index (Beck et al., 1988)

Physical conditions	

- Chronic diseases	LASA^a ^Questionnaire (Kriegsman et al., 1996)

- Pain	Chronic Graded Pain Scale (Von Korff & Miglioretti, 2005)

- Vision and hearing	LASA^a ^Questionnaires http://www.lasa-vu.nl/index.htm

- Sleep	Insomnia Rating Scale (Levine et al., 2003)

- Functional limitations	WHO-Disability Assessment Scale (Chwastiak et al., 2003)

- Medication use	Registration of medication use

- Smoking & drugs	Past & current smoking questions

- Alcohol dependency	Alcohol Use Disorders Identification Test (Babor et al., 1989)

- Physical Activities	International Physical Activities (Craig et al., 2003)

Biological determinants	

- Inflammation markers, BDNF^a ^& polymorphism	Fasting blood sample

- Autonomic Nervous System	Electrocardiography & impedance cardiography (VU-AMS; de Geus et al., 1995)

- Cortisol	6 saliva samples, including Dexamethasone Suppression Test (Wüst et al., 2000; APA 1986)

Psychol. determinants	

- Personality: Big Five	NEO-Five Factor Inventory (Costa & McCrae, 1995)

- Mastery	Pearlin Mastery scale (Pearlin & Schooler 1978)

- Daily Hassles	Worry Scale-R (Wisocki, 1986)

Social determinants	

- Early life events	NEMESIS questionnaire (De Graaf et al., 2004)

- Recent life events	Brugha Questionnaire (Brugha et al., 1985)

- Loneliness and affiliation	Loneliness and affiliation scale (De Jong Gierveld & Kamphuis, 1995)

- Social support	Close Person Inventory (CPI; Stansfeld et al., 1992)

Cognitive functioning	

- Global cognitive functioning	Mini-Mental State Examination (Folstein et al. 1975)

- Executive functioning	Stroop Color-Word test (short form) (Stroop 1935)

- Motivational problems	Digit Span (subtest WAIS^a^) (Wechsler, 1958)

- Memory function	Auditory Verbal Learning Test (Rey, 1964; van der Elst et al. 2005)

Use of Care	Trimbos/iMTA questionnaire for Costs associated with Psychiatric Illness (Hakkaart-van Roijen, 2002)

^a ^*SES *Social Economic Status; *LASA *Longitudinal Aging Study Amsterdam; *BDNF *brain-derived neurotrophic factor; *WAIS *Wechsler Adult Intelligence Scale

### Assessment of psychopathology

Diagnosis of depression and dysthymia according to DSM-IV-R criteria [48] is assessed with the Composite International Diagnostic Interview (CIDI; WHO version 2.1; life-time version). The CIDI is a structured clinical interview that is designed for use in research settings and has high validity for depressive and anxiety disorders. [9,50] As in NESDA, we added questions to determine the research DSM-IV diagnosis of current minor depression. Severity of depression was measured using a self-report questionnaire: the Inventory of Depressive Symptoms (IDS). [51] As co morbidity with anxiety disorders is high, anxiety disorders [General Anxiety Disorder (GAD), Panic Disorder (PAN), Agoraphobia (AGO) and Social Phobia (SOC)] were also assessed using the CIDI (12 month version). Severity of anxiety symptoms was measured using the Beck Anxiety Index (BAI), [52] a self-report questionnaire. Apathy which is assumed to be more common in late-life depression compared to early-life depression was assessed using the Apathy Scale as a self-report questionnaire [53].

### Vulnerability factors, stressors and protective factors

Demographic characteristics such as age, sex, ethnicity, place of living, household composition, and income were assessed with standard questions.

#### Psycho-social functioning

Childhood abuse, including emotional neglect as well as psychological, physical and sexual abuse, was assessed using a structured inventory. [54] Important life events during childhood and in the past years were assessed with the Brugha questionnaire. [55] Personality was assessed with the NEO-FFI, a 60 item questionnaire measuring the five personality domains neuroticism, extraversion, agreeableness, conscientiousness, and openness to experience [56] and a short mastery scale. [57] Daily hassles were assessed using a revised version of the Worry-Scale. [58] Details about present social support from the four most intimate persons were assessed with the Close Person Inventory, [59] and a self-report questionnaire on loneliness and affiliation [60].

#### Somatic and health markers

The presence of chronic diseases was assessed by means of a self-report questionnaire that has previously been used in NESDA. [47] The participants were asked whether they currently or previously had any of the following chronic diseases or disease events: cardiac disease (including myocardial infarction), peripheral atherosclerosis, stroke, diabetes mellitus, COPD (asthma, chronic bronchitis or pulmonary emphysema), arthritis (rheumatoid arthritis or osteoarthritis) or cancer, or any other disease. Compared to general practitioner information, the accuracy of self-reports of these diseases was shown to be adequate and independent of cognitive impairment [61].

Biomarkers that are parameters of cardiovascular status, immune function and metabolic syndrome were assessed in fasting blood. The interviews took place in the morning, and the respondents came in after an overnight fast. We drew 47.5 millilitres of blood which was immediately transferred to a local laboratory to start processing within an hour. Routine assays included assessment of haemoglobin, hematocrit, creatinine, total cholesterol, HDL and LDL cholesterol, glucose, triglycerides, albumin, thyroid stimulated hormone (TSH) and Free T4. The rest of the blood was processed and stored at −80°C for later assaying.

Systolic and diastolic blood pressures were measured twice in a supine position using an electronic Omron phygmomanometer. Doppler assessment of ankle and arm blood pressure allows calculation of the ankle/brachial index, which is an indicator of peripheral atherosclerosis [62].

Physical condition was assessed by means of measurement of handgrip using a hand-held dynamometer, which is an important indicator of physical frailty. Body composition assessment included objective, standardized assessments of height, weight and hip and abdominal circumference. Self reported functional limitations were assessed with the WHO-Disability Assessment Schedule. [63] Pain was assessed with the self report Chronic Graded Pain scale. [64] Sleep and insomnia was assessed with the Insomnia Rating Scale (IRS). [65] Vision and hearing were assessed with standard questions.

#### Physical stress regulation systems

The two most important biological stress regulation systems that have been hypothesized to be of importance in depression are the HPA-axis and the autonomic nervous system (ANS). Functioning of the HPA-axis was assessed by the use of cortisol concentrations in saliva, reflecting the free fraction of plasma cortisol, which was collected by the participants on two consecutive days. [66] Six saliva samples were taken: at the time of awakening (T1), 30 minutes post-awakening (T2), 45 minutes post-awakening (T3), 60 minutes post-awakening (T4) and at 22:00 h (T5). Dexamethasone suppression was measured by sampling the next morning at awakening (T6) after dexamethasone ingestion of 0.5 mg the night before (directly after T5). This Dexamethasone Suppression Test (DST) is a measure of HPA axis regulation and normally shows a decrease of morning cortisol concentrations due to inhibition of adrenocorticotropic hormone (ACTH) secretion after dexamethasone administration the night before. [67] The salivettes were restored in the tube labelled with date and time. After collecting all six samples, the subjects were asked to return the samples by post to the research centre. After receipt, salivettes were centrifuged at 2000 g for ten minutes, aliquoted and stored at −20°C.

The assessment of ANS functioning included heart rate, pre-ejection period and respiratory sinus arrhythmia, and was measured with the ambulatory recording system developed at the VU University in Amsterdam (VU-AMS, version 5 *fs*). Recording methodology of the VU-AMS as well as its reliability, validity and feasibility in large scale samples has been well documented [68,69].

#### Cognitive function

Tests for several cognitive domains were assessed: the Mini-Mental State Examination (MMSE) [70] for global cognitive functioning, the abbreviated version of the Stroop Colour-word test [71] for executive functioning; the subtest Digit Span from the Wechsler Adult Intelligence Scale [72] for motivational problems and a modified version of the Auditory Verbal Learning Test [73,74] for memory function.

#### Health behaviour

Assessment of health behaviour included smoking, alcohol use, physical activities and use of health care and medication. Smoking behaviour was assessed with standard questions, and the use of alcohol was assessed using the Alcohol Use Disorders Identification Test (AUDIT). [75] With the International Physical Activities Questionnaire (IPAQ) [76] energy expenditure based on sports and daily activities was calculated. Medical and psychological treatments and other professional support the persons have had for their mental and physical health problems was assessed with the Trimbos/iMTA questionnaire for Costs associated with Psychiatric Illness (TIC-P). [77] Medication use was determined by observation of the medication that the participants brought in.

### Longitudinal data collection

The course of late-life depression is followed up every 6 months by means of a postal assessment that includes questionnaires on the severity of depressive symptoms and somatic health in the past 6 months, incident (chronic) stressors and functional limitations, and use of medications and health care. The questionnaires are the same questionnaires that are used during the face-to-face assessments.

A second face-to-face assessment is being performed 2 years after the baseline assessment, and is currently going on. It consists of all measures (determinants and outcome variables) that are open to change, such as severity of psychopathology and diagnostics, change in socio-demographic characteristics, all somatic health and health markers, cognitive functioning, psycho-social functioning (except for the early life events and the personality questionnaire), and health behaviour. More detailed fluctuation of depressive symptoms during the past 2 years will be assessed with a Life Chart method. This instrument uses a calendar method to re-fresh memory and then assesses presence and severity of symptoms during each month in the past two years. [78] Collection of blood and VU-AMS registration are also repeated.

The follow-up assessment lasts about 3 hours and the same instruments will be used as in the baseline assessment (see Table 1), including written questionnaires, an interview, and a medical examination. A third follow-up face-to-face assessment is planned 6 years after the baseline assessment.

### Quality of the data

All interviews and physical examinations are conducted by carefully selected research assistants, mainly consisting of psychologists and mental health care nurses. The research assistants received a five day training from an experienced and certified trainer before the baseline and follow-up assessments. The research assistant is certified to conduct assessments after approval of two complete interviews which were audio taped and judged by the trainer. Question wording and probing behaviour of interviewers will frequently be monitored by checking a random selection of about 10% of all taped interviews. Data are collected with Computer Assisted Personal Interviewing (CAPI) procedures on laptop computers and with self-administered questionnaires.

### Data management and control

Data management of NESDO is coordinated at the Department of Psychiatry of the VUMC. The collected data from the different sites are send to the coordinating centre, where the data are entered in the central database. A special team of data managers takes care of data quality, data archiving and the creation of variables and scales. Data quality check procedures are in place and are routinely carried out to review missing data and check for inconsistencies.

### Ethical issues

The study protocol of NESDO has been approved centrally by the Ethical Review Board of the VU University Medical Center, and subsequently by the local ethical review boards of the Leiden University Medical Center, University Medical Center Groningen and the Radboud University Medical Center in Nijmegen. Before participating in the study, all persons were provided with oral and written information. Written informed consent was obtained from all participants at the start of the baseline assessment. Written informed consent was asked for participating in the study, for permission to use genetic information, to retrieve medical information from the GP's, and to link information to external databases. A privacy protocol has been developed in which confidentiality of data is guaranteed by using a unique research ID number for each respondent, which enables to identify individuals without using their names. Only the data manager has access to the record that links the ID number with the name of the participant.

## Results

The baseline sample consists of 378 depressed persons and 132 non-depressed controls. The overall sample has a mean age of 70.6 years (SD: 7.3; range = 60-93) and consists of 331 (64.9%) women and 179 (35.1%) men. The average years of education is 11.0 (SD = 3.6; range 5-18 years). The majority of the sample has the Dutch nationality (99.4%).

The depressive persons do not differ from the non-depressed controls with respect to mean age and sex, but they have a lower level of education, are more often divorced or widowed, and have a lower score on the MMSE (Table 2). From the depressed persons 95% has a current (six-month) major depressive disorder, 26.5% has dysthymia and 5.6% has a minor depression. 26.5% of the depressed persons have two depressive disorders, major depressive disorder and dysthymia (Table 3). Mean age of onset of the depressive disorders was around 49 years. About thirty percent of the persons with major depression (MDD) and dysthymia had an onset before the age of 40 years, whereas about 33% of the persons with MDD and 41% of the persons with dysthymia had an onset after 60 years. For 33.1% of the depressed persons it was their first episode. Severity of the depression symptoms varied between mild and very severe. Forty-one percent of the depressed persons had in the past 6 months also an anxiety disorder.

**Table 2:** Characteristics of the sample (*N = *510)

	Depressed persons (*n = *378)	Non-depressed controls (*n = *132)	p
Age, mean (SD)	70.7 (7.4)	70.1 (7.2)	0.37

- 60-69, n (%)	189 (50.0)	74 (56.1)	0.48

- 70-79, n (%)	132 (34.9)	40 (30.3)	

- > 80, n (%)	57 (15.1)	18 (13.6)	

Female, n (%)	250 (66.1)	81 (61.4)	0.34

Level of education			< 0.001

- Low, n (%)	78 (20.6)	9 (6.8)	

- Middle, n (%)	221 (58.5)	71 (53.8)	

- High, n (%)	79 (20.9)	52 (39.4)	

Marital status			0.001

- Never been married, n (%)	30 (7.9)	10 (7.6)	

- Married, n (%)	174 (46.0)	86 (65.2)	

- Living apart, n (%)	16 (4.2)	0	

- Divorced, n (%)	54 (14.3)	10 (7.6)	

- Widow(er), n (%)	104 (27.5)	26 (19.7)	

Cognitive function, mean MMSE^1 ^(SD)	27.69	28.34	0.001

^1 ^MMSE: Mini-Mental State Examination

**Table 3:** Characteristics of depression and co morbid anxiety disorders from the depressed cohort (*N *= 378)

Major depression	
Major depression, current^1^, n (%)	359 (95.0)

Mean age of onset (SD)	48.6 (20.3)

Age of onset in categories (%)	

- < 40 yrs	30.6

− 40-60 yrs	34.5

− ≥ 60 yrs	32.9

- No answer	1.9

**Dysthymia**	

Dysthymia, current^1^, %	100 (26.5)

Mean age of onset (SD)	50.7 (19.7)

Age of onset in categories, %	

− < 40 yrs	29.3

− 40-60 yrs	29.3

− ≥ 60 yrs	41.4

- No answer	1.0

**Minor depression (current), %**	21 (5.6)

**Severity depressive symptoms**	

Mean IDS score^2 ^(SD)	30.14 (13.0)

IDS^2 ^categories	

- mild	37.5

- moderate	35.4

- severe/very severe	27.1

**First episode, %**	33.1

**Comorbid anxiety disorders^1^**	

Any anxiety disorder, n (%)	155 (41.0)

Social Phobia, n (%)	74 (19.6)

Panic disorder, without agoraphobia, n (%)	29 (7.7)

Agoraphobia, n (%)	41 (10.8)

Generalized Anxiety Disorder, n (%)	40 (10.6)

^1 ^Current: disorder in the past 6 months

^2 ^Assessed with Inventory of Depressive Symptomatology (IDS)

## Discussion

NESDO is a multi-site naturalistic cohort study that offers us the possibility to examine the determinants, the course and consequences of depressive disorders in older persons, and to compare these with those of depression earlier in adulthood. Because of the increase of the aging population in the near future, the number of older persons suffering from (chronic) depression will further grow in the forthcoming years, and thus will become an even more relevant issue for public health care. It is of great importance to uncover mechanisms responsible for its chronic course and co morbidity. Differences in (the determinants of) the course and consequences of depression have seldom been directly compared between older and younger adults using a similar study design and with comparable instruments. Knowledge about the adequacy of the postulated differences between late-life and early-life depression is important, since it will yield important implications for mental health care practice and research. By combining data from NESDA and NESDO we will be able to address important questions that have potential relevance to the development of interventions capable of improving the course of late-life depression. The clinical relevance of the study is enhanced by the fact that several determinants of the course of depression may be used in prevention and treatment programs, which may be implemented in regular care.

## Conclusions

The NESDO sample offers the opportunity to study the (determinants) of the long-term course of depression in older persons, and to pool data with NESDA. This provides us with a large (longitudinal) database of clinically depressed persons with adequate power and a large set of neurobiological, psychosocial and physical variables from both younger and older depressed persons.

## Competing interests

The authors declare that they have no competing interests.

## Authors' contributions

All authors are key members of the research consortium and have made substantial contributions to the conception and the design of the study, and acquisition of the baseline data. They have all been involved in writing the manuscript and have given their approval for the final version.
